# Impact of Preoperative Total Proteins and Glycated Hemoglobin on Recurrences after Early Colorectal Cancer

**DOI:** 10.3390/nu13020711

**Published:** 2021-02-23

**Authors:** María-José Castro, José-María Jiménez, María López, María-José Cao, Jair Santos-Torres, Alberto López, Ana Moreno, Jaime Ruiz-Tovar

**Affiliations:** 1 Faculty of Nursing, University of Valladolid, 47005 Valladolid, Spain; mariajose.castro@uva.es (M.-J.C.); maria.lopez.vallecillo@uva.es (M.L.); mjcao@enf.uva.es (M.-J.C.); ajumor@yahoo.es (A.M.); 2 Department of Surgery, Dr Sulaiman AlHabib Hospital, Alkhobar 34423, Saudi Arabia; jairsantost@yahoo.es; 3 Department of Surgery, Hospital de Mieres, 33611 Mieres, Asturias, Spain; albertolopezd@hotmail.com; 4 Department of Surgery, Hospital Severo Ochoa, 28911 Leganés, Madrid, Spain; 5 Department of Surgery, Rey Juan Carlos University, 28933 Móstoles, Madrid, Spain; jruiztovar@gmail.com

**Keywords:** early colorectal cancer, outcome, total proteins, glycated hemoglobin

## Abstract

Background: The outcome of colorectal cancer is mostly based on TNM classification. There are several factors determining that patients with the same tumoral stage present different outcomes. The nutritional status has been related to the immunological response and may affect the oncologic results. The purpose of this study was to determine if preoperative nutritional parameters may predict the oncologic outcome in patients with early colorectal cancer. Methods: A prospective observational study of patients undergoing elective surgery for colorectal cancer was performed with stage I. Preoperative nutritional assessment included glycemic and lipid profiles, total proteins, and albumin levels. These parameters were correlated with tumoral recurrence during a follow-up of at least 24 months. Results: During the period of study, 744 patients were operated on and 228 (30.6%) followed the inclusion criteria for this study. Recurrence rate was 5.7% (13 patients). Patients with hypoproteinemia showed a 7.8-fold greater risk of recurrence during the first 24 months after surgery [OR 7.8 (CI95% 1.3–48), *p* = 0.012]. Patients with glycated hemoglobin levels (HbA1c) > 6.2% showed a 2.3 increased risk of recurrence [OR 2.3 (CI95% 1.1–4.7; *p* = 0.01]. Conclusions: Preoperative values of total proteins and HbA1c correlate with the recurrence rate in early colorectal cancer.

## 1. Introduction

Colorectal cancer (CRC) represents 10–15% of all new cases of neoplastic disease arising every year in developed countries. In USA, 150,000 new cases are diagnosed yearly, estimating that nearly one third will die from this in the next 5 years, despite having undergone a resective surgery with curative aims [1]. Global mortality is calculated to be around 31.7 and 24.7 per 100,000 inhabitants/year in males and females, respectively. Thus, CRC represents one of the main sociosanitary problems in the actual society [2]. 

Around 15% of the CRC cases will present distant metastases at the time of diagnosis, and 50% of the patients with locally advanced disease will develop metachronous metastases, leading to mortality in less than two years of follow-up, despite adequate surgical and adjuvant treatment [3].

The outcome of CRC is determined by diverse pathologic, clinic, and biological factors. Among them, the tumoral stage (TNM classification) has shown to be the most accurate factor to determine the prognosis of the patients with CRC [4]. Early stages (Stage 1) are associated with better prognosis, with a 5-year survival rate close to 90% [4]. 

However, there are several factors determining that patients with the same tumoral stage present different outcomes. Among them, the preoperative nutritional status has been associated with the long-term oncologic prognosis, in terms of recurrence of mortality [5]. 

Malnutrition has been widely associated with early postoperative complications, such as leakage and surgical-site infections. Altogether, it affects the immunological response and may impair the oncologic results [6]. 

The aim of this study was determine if preoperative nutritional parameters may predict the oncologic outcome in patients with early colorectal cancer.

## 2. Materials and Methods

A prospective observational study was conducted between January 2013 and January 2015, including patients diagnosed of early colorectal cancer (stage I) and operated with curative aims. Patients with immunological disorders and with criteria of malnutrition were excluded. Malnutrition was defined following the ESPEN criteria (weight loss > 10–15% of total body weight during the last 6 months, BMI < 18.5 kg/m^2^, albumin levels < 3 g/dL without liver or renal causes) [7]. Undernourished patients were evaluated referred to the Endocrinology Department to start nutritional supplementation for 14 days before surgery. These patients were excluded from analysis, as nutritional supplementation may represent a bias in the results. Patients died due to postoperative complications were also excluded.

The follow-up scheme consisted of visits to the Outpatient Clinic every 3 months during the first 2 years. Then, there were visits every 6 months until 5 years of follow-up. During these visits, the patients underwent physical examination. In addition, blood simple were obtained at all the visits, assessing hemogram, biochemical parameters, and tumoral markers. Thorax-abdomen-pelvic CT scan was conducted every 6 months and colonoscopy every year during the follow-up period. The follow-up was initially performed by the colorectal surgeon. In cases of suspicion of the recurrence of disease, the patients were evaluated by the Oncology Multidisciplinary Committee, composed of surgeons, medical and radiotherapeutic oncologists, radiologists, endoscopists, and pathologists, in order to determine the most appropriate treatment for the patients at any time. 

Preoperative blood sample was obtained 14 days before surgery after a minimal fasting period of 8 hours. Antidiabetic or hypolipemiant drugs were not stopped before undergoing this blood sample.

Analyzed variables included the preoperative glycemic profile (fasting glucose and glycated hemoglobin (A1c)), lipid profile (total cholesterol, HDL-cholesterol, LDL-colesterol and triglycerids), total proteins, and albumin levels. Postoperative complications, recurrence, and mortality rates for the first 2 years of follow-up were recorded.

Statistical analysis was performed with SPSS 22.0 software. Means were compared with the Student´s *t*-test (Mann–Whitney test in nonparametric variables). The Kaplan–Meier method was used for the survival and recurrence analysis. The prognostic ability of the biochemical parameters was evaluated by a receiver operator characteristic curve. The area under the curve (AUC) was given with 95% CI, and the cutoff point was calculated maximizing the sensitivity in accordance with the Youden index. Recurrence rate was calculated using Kaplan–Meier curves for the determined cut-off points. A *p*-value < 0.05 was considered to be statistically significant.

## 3. Results

During the period of study, 744 patients were operated for CRC, but only 228 (30.6%) followed the inclusion criteria for this study (stage I) and did not present exclusion criteria. There were 139 (61%) males and 89 (39%) females, with a mean age of 69.8 ± 11.4 years. Comorbidities included Diabetes Mellitus 28.9% (*n* = 66), Hypertension 46.5% (*n* = 106), dyslipidemia 29.8% (*n* = 68), cardiopathy 19.3% (*n* = 44), and chronic obstructive pulmonary disease 1.7% (*n* = 4). Sixty patients with diabetes mellitus were under treatment with oral antidiabetic drugs and 6 under insulin therapy (Table 1). 

The surgical procedures are described in Table 2. All of the procedures were initially laparoscopically performed, with a conversion rate of 4.4% (10 patients). Curative resection was performed in all the cases.

Postoperative complications are summarized in Table 3. Ten patients (4.4%) required another operation, including all the patients presenting anastomotic leak and one intraoperative bleeding. Median hospital stay was five days (range 4–56 days).

### 3.1. Histological Examination

All the specimens were adenocarcinomas. Mean tumoral size was 1.7 ± 2.4 cm and the mean number of resected lymph nodes was 10.1 ± 3.9, all of them without metastases (N0). Local stage was T1 in 105 patients (46.1%) and T2 in 123 (53.9%).

### 3.2. Follow-Up

A minimal follow-up of 24 months was established, a median of 34 months (range 24–59 months). Recurrence rate was 5.7% (13 patients), including nine liver metastases, three lung metastases and one case with metastases in multiple locations. Tumoral staging was T2N0 in all the cases presenting recurrences. Mortality during the first two years postoperatively appeared in only two patients (0.88%).

### 3.3. Correlation between Analytical Parameters and Recurrence

Preoperative nutritional, glycemic, and lipid profile data are shown in Table 4.

### 3.4. Total Proteins

**Figure 1:**
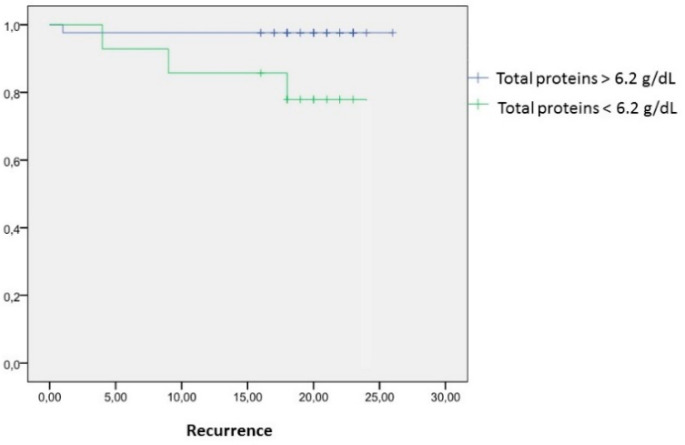
Kaplan–Meier curve between preoperative total protein levels and recurrence after 24 months of follow-up.

Mean total protein values within the patients presenting recurrence were 6 ± 0.3 g/dL versus 6.8 ± 0.6 g/dL in disease-free patients (mean difference 0.8 g/dL (CI95% (0.09–1.33); *p* = 0.023). A cut-off point was established at 6.2 g/dL (OR 7.8 (CI95% (1.3-48); *p* = 0.012) for protein values below 6.2 g/dL. Recurrence rate was 16.7% and 22.2% at one and two years of follow-up, respectively, in those patients with protein values below 6.2 g/dL, whereas the recurrence rate was 0.6% at one and two years after surgery, when protein levels overcome 6.2 g/dL. Patients with total protein levels > 6.2g/dL have an increased risk for presenting tumoral recurrences after 24 months of follow-up (RR 35.9; CI95% (12.8–94.6; *p* < 0.001) (Table 5 and Figure 1).

### 3.5. Glycated Hemoglobin (A1c)

**Figure 2:**
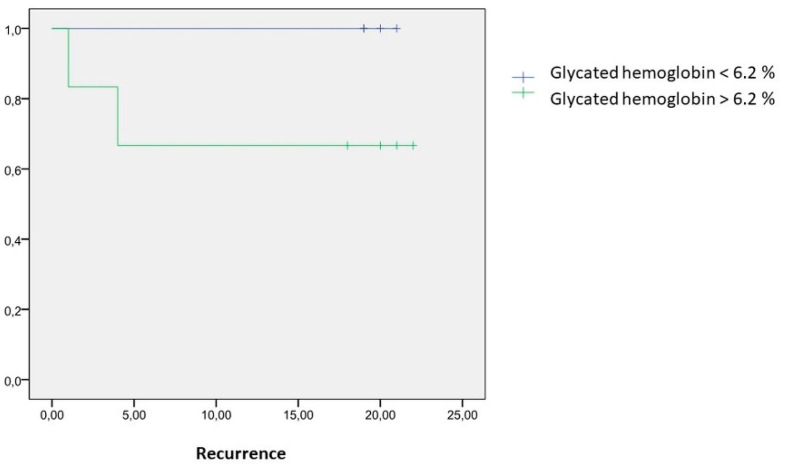
Kaplan–Meier curve between preoperative glycated hemoglobin levels and recurrence after 24 months of follow-up.

Mean A1c values within the patients presenting recurrence was 6.8 ± 2.8% versus 6.1 ± 6.4 in disease-free patients (mean difference 0.7% (CI95% (0.2–1.5%); *p* = 0.009). A cut-off point was established at 6.2% (OR 2.3 (CI95% (1.1–4.7); *p* = 0.01). Recurrence rate was 31.7% at one and two years of follow-up, respectively, in those patients with A1c levels over 6.2%, whereas there were no recurrences at one and two years after surgery, when A1c levels were below 6.2%. Patients with A1c values > 6.2% have an increased risk for presenting tumoral recurrences after 24 months of follow-up (RR 62.9; CI95% (24.6–133.9); *p* < 0.001) (Table 6 and Figure 2).

Fasting glucose levels within patients presenting recurrence tends to be higher than in disease-free patients (128.1 ± 24.3 mg/dL vs. 106.9 ± 21.2 mg/dL; *p* = 0.09).

Significant correlations between the rest of the nutritional parameters and a glycemic profile with a recurrence rate could not be observed.

## 4. Discussion

Nutritional status has been associated with the oncologic outcome in diverse neoplasms. These are mostly related to the immunologic and inflammatory response. The ability of tumoral cells for local invasion and development of distal metastases depend on the intrinsic features of neoplastic cells and the adjacent environment [8]. The altered phenotype of tumoral cells together with the tissue destruction they cause stimulate the inflammatory response in the adjacent parenchyma. This inflammatory reaction implies a great expenditure of energy and leads to the development of a systemic catabolic status to increase the availability of glucose and other energetic precursors [9].

Malnutrition is often a consequence of the constitutional syndrome associated with malignant neoplasms. Thus, patients present malnutrition in any degree quite frequently at the time of diagnosis. Therefore, a nutritional evaluation is mandatory before setting the indication for surgical treatment and nutritional optimization is recommended prior to the operation [10]. There is increasing evidence that malnutrition is associated with lower survival rates in diverse neoplasms [11,12,13]. Despite most studies including all tumoral stages in their survival analysis, it has been also demonstrated that malnutrition hinders the oncologic outcome even in early stages of gastrointestinal tumors [14].

Serum proteins are an indirect marker of the nutritional status of the patients. Their determination is easy and widely available in routine clinical practice. Albumin is the main component of serum proteins, and it is considered a prognostic indicator for major surgery and oncologic outcome after diverse neoplasms [15,16,17]. Low albumin levels in oncologic disease might be secondary to the intense systemic inflammatory response syndrome, which requires an increased synthesis of acute-phase proteins, reducing the synthesis of other proteins, such as albumin. When this process lasts for a long period of time, this leads to a protein depletion in all of the organisms, which reduces the immunologic resistance and contributes to early death [17].

In the present study, we observed a correlation between preoperative total protein levels below 6.2 g/dL and disease recurrence. Curiously, we established a cut-off point at a protein level within the normal range. However, it has been widely demonstrated that preoperative normo-nourished patients can develop undernutrition during the postoperative course, secondary to the inflammatory response to the surgical damage and a delay in the reintroduction of oral nutrition [18]. Therefore, the actual recommendations of perioperative care within Enhanced Recovery After Surgery protocols consider an eventual indication of preoperative oral supplementation in normo-nourished patients undergoing colorectal surgery, in order to reduce postoperative complications and a certain degree of immunologic deficiency associated with the postoperative decrease of serum proteins [19]. 

In our study, we also observed that patients with preoperative A1c levels over 6.2% present a 2.3 times higher risk of tumoral recurrence 24 months after surgery. Despite the fact that we failed to demonstrate significantly different levels in fasting glucosa, the patients with recurrence tend to show more elevated glucosa levels. In conclusion, diabetic patients are more prone to show tumoral recurrence of early colorectal cancer.

Type 2 diabetes is characterized by an insulin resistence and hiperinsulinemia. Diverse in vitro and in vivo studies have shown that insulin and Insulin-like Growth Factor (IGF-1) have mitogenic effects on colorectal cancer cells, stimulating cell proliferation and inhibiting apoptosis [20,21,22]. In addition, epidemiological studies have determined a positive association between insulin and C peptide with an increased risk of colorectal cancer [23]. Moreover, the hyperglycemia itself also promotes carcinogenesis, as glycolysis is an essential step for the energetic metabolism of the tumor, generating oxidative stress [24,25,26]. Several cohort studies have correlated elevated serum glucosa levels with an increased risk of developing different neoplasms [27,28,29].

Though this is the first study reporting associations between A1c and oncologic outcome in patients with early colorectal cancer, hyperglycemia and the diagnosis of diabetes mellitus have been involved with a certain immunosupression status, which may favor a tumoral progression [28]. In addition, uncontrolled hyperglycemia is a risk factor for surgical complications, mostly infectious ones. The development of complications has also been related with a progression of the disease. Consequently, it seems to be advisable to optimize the control of diabetes preoperatively, but also to establish strict control after surgery [30,31].

One of the main limitations of the present study is the small number of relapses, which makes the interpretation and extrapolation of the results difficult. Additional studies and multiple centers and with greater sample size are necessary to confirm our results.

## 5. Conclusions

Total protein levels below 6.2 g/dL and A1c values over 6.2% are associated with increased recurrence risk in early colorectal cancer. Further studies must evaluate if a preoperative optimization of these parameters would have a positive impact on the oncologic outcome.

## Figures and Tables

**Table 1:** Patients’characteristics.

	Disease-Free (*n* = 215)	Recurrence (*n* = 13)
Age, mean (SD) 69.8 (11.4)	69.7 (11.1)	10.7 (13.8)
Males/females, *n*(%) 139/89 (61/39)	131/84 (60.9/39.1)	8/5 (61.5/38.5)
Diabetes mellitus, *n*(%) 66 (28.9)	62 (28.8)	4 (30.8)
Hypertension, *n*(%) 106 (46.5)	100 (46.5)	6 (46.2)
Dyslipidemia, *n*(%) 68 (29.8)	64 (29.8)	4 (30.8)
Cardiopathy, *n*(%) 44 (19.3)	41 (19.1)	3 (23.1)
COPD, *n*(%) 4 (1.7)	4 (1.9)	0

COPD: Chronic obstructive pulmonary disease.

**Table 2:** Surgical techniques.

Surgical Technique	Disease-Free (*n* = 215)	Recurrence (*n* = 13)
Left colectomy, *n*(%) 39.5	(89) 41.4%	5 (38.5%)
Right colectomy, *n*(%) 25.5	(58) 27%	4 (30.8%)
Low anterior resection of rectum, *n*(%) 24.2	(54) 25.1%	4 (30.8%)
Miles procedure, *n*(%) 8.7	(19) 8.8%	0
Total colectomy, *n*(%) 2.1	(5) 2.3%	0

**Table 3:** Postoperative complications.

	Disease-Free (*n* = 215)	Recurrence (*n* = 13)
Incisional SSI, *n*(%) 14 (6.1)	12 (5.6)	2 (15.4)
Organ-space SSI, *n*(%) 6 (2.6)	5 (2.3)	1 (7.7)
Postoperative bleeding, *n*(%) 4 (1.8)	4 (1.9)	0
Anastomotic leak, *n*(%) 9 (3.9)	7 (3.3)	2 (15.4)

**Table 4:** Preoperative nutritional, glycemic, and lipid profile.

Variable	Disease-Free (*n* = 215)	Recurrence (*n* = 13)	*p*-Value
Glucose (mg/dL)	106.9 + 21.22	128.1 + 24.3	0.085
Glycated hemoglobin (%)	6.0 ± 0.31	6.8 + 0.6	0.009
Albumin (g/dL)	4.23 ± 0.23	4.03 + 0.58	0.378
Total proteíns (g/dL)	6.8 ± 2.8	6.1 + 6.4	0.023
Triglycerids (mg/dL)	104.93 ± 13.74	109.9 + 18.5	0.582
Total cholesterol (mg/dL)	173.01 ± 14.03	175.5 + 21.3	0.634
HDL cholesterol (mg/dL)	48.01 ± 11.52	46.9 + 14.2	0.512
LDL cholesterol (mg/dL)	118.62 ± 12.33	121.1 + 16.8	0.585

**Table 5:** Distribution of patients with recurrence depending on total protein levels two years after surgery.

*n* = 228	Total Proteins > 6.2g/dL	Total Proteins < 6.2g/dL
Recurrence	1	12
Disease free	173	42

**Table 6:** Distribution of patients with recurrence depending on A1c levels two years after surgery.

*n* = 228	A1c > 6.2%	A1c < 6.2%
Recurrence	13	0
Disease free	28	187
